# Targeting the TLR2 Receptor With a Novel Thymopentin-Derived Peptide Modulates Immune Responses

**DOI:** 10.3389/fimmu.2021.620494

**Published:** 2021-05-27

**Authors:** Xubiao Wei, Lulu Zhang, Rijun Zhang, Rujuan Wu, James N. Petitte, Yanfei Hou, Dayong Si, Baseer Ahmad, Henan Guo, Manyi Zhang, Qiang Cheng, Yucui Tong

**Affiliations:** ^1^ Laboratory of Feed Biotechnology, State Key Laboratory of Animal Nutrition, College of Animal Science and Technology, China Agricultural University, Beijing, China; ^2^ Prestage Department of Poultry Science, College of Agriculture and Life Sciences, North Carolina State University, Raleigh, NC, United States; ^3^ School of Pharmaceutical Sciences, Tsinghua University, Beijing, China

**Keywords:** TP5, immunopotentiating activity, physiological stability, molecular dynamics simulations, TLR2 cluster, NF-κB signaling

## Abstract

The innate and adaptive immune systems act in concert to protect us from infectious agents and other harmful substances. As a state of temporary or permanent immune dysfunction, immunosuppression can make an organism more susceptible to infection, organ injury, and cancer due to damage to the immune system. It takes a long time to develop new immunomodulatory agents to prevent and treat immunosuppressive diseases, with slow progress. Toll-like receptor 2 (TLR2) agonists have been reported as potential immunomodulatory candidates due to their effective activation of immune responses. It has been demonstrated that thymopentin (TP5) could modulate immunity by binding to the TLR2 receptor. However, the fairly short half-life of TP5 greatly reduces its pharmacological potential for immunosuppression therapy. Although peptide cathelicidin 2 (CATH2) has a long half-life, it shows poor immunomodulatory activity and severe cytotoxicity, which seriously hampers its clinical development. Peptide hybridization is an effective approach for the design and engineering of novel functional peptides because hybrid peptides combine the advantages and benefits of various native peptides. In this study, to overcome all these challenges faced by the parental peptides, six hybrid peptides (CaTP, CbTP, CcTP, TPCa, TPCb, and TPCc) were designed by combining the full-length TP5 with different active fragments of CATH2. CbTP, the most potent TLR2 agonist among the six hybrid peptides, was effectively screened through in silico analysis and *in vitro* experiments. The CbTP peptide exhibited lower cytotoxicity than either CATH2 or TP5. Furthermore, the immunomodulatory effects of CbTP were confirmed in a CTX-immunosuppressed mouse model, which showed that CbTP has increased immunopotentiating activity and physiological stability compared to the parental peptides. CbTP successfully inhibited immunosuppression and weight loss, increased immune organ indices, and improved CD4^+^/CD8^+^ T lymphocyte subsets. In addition, CbTP significantly increased the production of the cytokine TNF-α and IL-6, and the immunoglobulins IgA, IgM, and IgG. The immunoenhancing effects of CbTP were attributed to its TLR2-binding activity, promoting the formation of the TLR2 cluster, the activation of the TLR2 receptor, and thus activation of the downstream MyD88-NF-кB signaling pathway.

## Introduction

The occurrence and development of the animal immune system is a lengthy process, and it has been formed, developed and perfected gradually during long-term evolution and adaptation to biological development (1, 2). The innate and adaptive immune systems act in concert to protect us from infectious agents and other harmful substances (1, 2). As a state of temporary or permanent immune dysfunction, immunosuppression can make an organism more susceptible to infection, organ injury, and cancer due to damage to the immune system (3). It takes a long time to develop new immunomodulatory agents to prevent and treat immunosuppressive diseases, with slow progress. Therefore, it is necessary to explore and develop a new immunomodulatory agent to prevent and treat immunosuppressive diseases.

Toll-like receptors (TLRs) are a family of pattern recognition receptors that recognize conserved structures associated with infection or tissue damage (4, 5). TLRs also play a fundamental role in the development of adaptive immunity and the regulation of immune responses (6–8). TLR2, an important signal transduction-associated membrane molecule, is widely expressed by a variety of cells in many animal species (5, 9). Activation of TLR2 initiates the recruitment of the adapter molecules TIRAP, MyD88, IRAKs, and TRAF6, subsequent activation of MAPK, IKK and NF-κB, and increased expression of their respective target genes, including inducible nitric oxide synthase (iNOS), TNF-α, and IL-6 (4, 6). Therefore, TLR2 agonists are promising candidates as vaccine adjuvants and pharmaceuticals that support immunotherapies since they can directly activate TLR2 and thereby enhance both humoral and cellular immune responses.

Thymopentin (TP5), a pentapeptide corresponding to position 32-36 of thymopoietin, exhibits biological activity responsible for phenotypic differentiation of T cells and the regulation of immune systems (10, 11). TP5 has been clinically used for the treatment of patients with immunodeficiency diseases, such as rheumatoid arthritis, cancers, hepatitis B virus infection, and acquired immunodeficiency syndrome (12, 13). Furthermore, it has been demonstrated that TP5 can enhance the immune response by binding to the TLR2 receptor (14). Overall, TP5 is used in the treatment of immunodeficiencies, malignancies, and infections due to its immunoregulatory activity and low cytotoxicity (10–14).

Cathelicidin 2 (CATH2), a highly cationic chicken heterophil-derived peptide, exhibits great potential in immune regulation (15–18). C has been shown to be involved in phagocytosis and neutralization of lipopolysaccharides (LPS) or lipoteichoic acid (LTA) during TLR stimulation (15, 19). Additionally, CATH2 can activate the expression of chemokines [e.g., monocyte chemotactic protein 1 (MCP-1)] for recruitment of immune cells and induces the secretion of cytokines such as tumor necrosis factor alpha (TNF-α), interleukin-6 (IL-6), and IL-1β from monocytes, macrophages and dendritic cells, thereby triggering activation of the immune responses (15–19). Therefore, CATH2 could be developed into an immune-enhancing agent to attenuate or treat immunosuppression.

TP5 is a potent immunopotentiator and plays an important role in the process of immune enhancement. However, the fairly short half-life of TP5 greatly reduces its pharmacological potential for immunosuppression therapy (20–22). Although CATH2 has a long half-life, it shows relatively limited immunoregulatory activity and some cytotoxicity toward eukaryotic cells, which seriously hampers its clinical development (15). As an effective strategy which could combine the advantages of different native peptides, hybridization has been put forward recently to obtain a new peptide with more ideal biological activity but minimal cytotoxicity (23, 24). As previously reported, CATH2 (4–21), CATH2 (1–13), and CATH2 (5–15) exhibit some immunoregulatory potential (15, 16, 25). In the present study, we designed six hybrid peptides, CATH2 (4–21)-TP5 (CaTP), CATH2 (1-13)-TP5 (CbTP), CATH2 (5-15)-TP5 (CcTP), TP5- CATH2 (4-21) (TPCa), TP5- CATH2 (1-13) (TPCb), and TP5- CATH2 (5-15) (TPCc), by combining the three different active fragments of C with full-length TP5 in different arrangements. We hypothesized that in such constructs, the immunoregulatory properties and physiological stability of the native peptides would be conserved or elevated, and the cytotoxicity would be reduced. The hybrid peptides were screened by molecular docking test with TLR2 and *in vitro* experiments based on their immunoregulatory activity and cytotoxicity. We further investigated whether the hybrid peptide could provide effective therapy against cyclophosphamide (CTX)-induced immunosuppression and explored the underlying mechanisms, using an *in vitro* model and a mouse model of immunosuppression.

## Materials and Methods

### Designing the Hybrid Peptide

Six hybrid peptides, CaTP, CbTP, CcTP, TPCa, TPCb, and TPCc, were designed by combining the three different active fragments of C with full-length TP5 in different arrangements. The complete amino acid sequences of the hybrid peptides and their parental peptides are listed in **Table 1**. The secondary structure of the peptides were analyzed using ProtParam (ExPASy Proteomics Server: http://www.expasy.org/tools/protparam.html). The three-dimensional (3D) structures of CaTP, CbTP, CcTP, TPCa, TPCb, and TPCc peptides were constructed by I-TASSER (http://zhanglab.ccmb.med.umich.edu/I- TASSER/).

**Table 1:** Design and sequences of the peptides.

Peptides	Sequence
TP5	RKDVY
Cathelicidin-2 (CATH2)	RFGRFLRKIRRFRPKVTITIQGSARFG
CATH2 (4-21)-TP5 (CaTP)	RFLRKIRRFRPKVTITIQRKDVY
CATH2 (1-13)-TP5 (CbTP)	RFGRFLRKIRRFRRKDVY
CATH2 (5-15)-TP5 (CcTP)	FLRKIRRFRPKRKDVY
TP5-CATH2 (4-21) (TPCa)	RKDVYRFLRKIRRFRPKVTITIQ
TP5-CATH2 (1-13) (TPCb)	RKDVYRFGRFLRKIRRFR
TP5-CATH2 (5-15) (TPCc)	RKDVYFLRKIRRFRPK

### Molecular Docking

The constructed 3D structure modes of the hybrid peptides were subjected to molecular docking and visualized by PYMOL software. The crystal structure of TLR2 was generated through the Protein Data Bank (PBD ID: IFYW). For protein-peptide docking, Zdock 3.0.2 was employed to acquire the initial complex structure of the TLR2-hybrid peptide complex. Altogether, 3,600 decoy structures were obtained from the Zdock binding prediction, from which the best decoy structure with the lowest energy was chosen for the following flexible docking analysis. A total of 1,000 docking runs of each molecule were performed by flexpepdock (http://flexpepdock.furmanlab.cs.huji.ac.il/), among which the plausible TLR2-hybrid peptide docking structure with the lowest interface binding energy score was chosen to screen the hybrid peptides.

### Peptide Synthesis

The hybrid peptides CaTP, CbTP, CcTP, TPCa, TPCb, and TPCc and their parental peptides CATH2 and TP5 were synthesized (95% purity) by KangLong Biochem Ltd (Jiangsu, China) and their molecular weights were determined by matrix-assisted laser desorption/ionization time-of-flight mass spectrometry (MALDI-TOF-MS). The peptides were synthesized in free C-terminal acid form. The peptides were dissolved in endotoxin-free water and stored at –80°C until analysis.

### Cell Culture

Murine macrophage cells (RAW264.7) were purchased from the Shanghai Cell Bank, the Institute of Cell Biology, China Academy of Sciences (Shanghai, China). The cells were cultured in DMEM (HyClone) supplemented with 10% (v/v) fetal bovine serum (FBS; Bioscience) and 1% (v/v) penicillin/streptomycin (HyClone) and maintained at 37°C in a fully humidified 5% CO_2_ incubator.

### Cell Proliferation Assay

The Cell Counting Kit-8 (CCK-8) Assay Kit (Dojinbo, Japan) was used to detect the effect of peptides on cell proliferation. Briefly, 1 × 10^4^ cells/well were seeded in 96-well culture plates, and these cells were then incubated with CCK8 reagent for 4 h at 37°C at the 24 h time point. The absorbance of each well was measured at 450 nm using a 96-well plate reader. Experiments were repeated five times. Cell viability was evaluated by using the following equation:

$$Cell\;viability\;(\%)=\left(\frac{\text{OD}450(\text{sample})}{\text{OD}450(\text{control})}\right)\times 100\%,$$

where OD_450_ (sample) is the absorbance of the peptide-treated cells at 450 nm and OD_450_ (Control) is the absorbance of the control (nonpeptide-treated cells) at 450 nm.

### Immunomodulatory Activity in RAW264.7 and THP-1 Cell Line

RAW264.7 cells and human THP-1 monocyte-macrophage cells were treated with or without 10 μg/mL peptides for 24 h at 37°C. Levels of tumor necrosis factor (TNF)-α, interleukin (IL)-6 and IL-1β in the cell culture supernatant were qualified using commercial enzyme-linked immunosorbent assay (ELISA) kits (eBioscience, San Diego, USA). Samples were assessed according to the manufacturers’ instructions.

### *Ex Vivo* Stability of CbTP in Human Serum

Human serum was purchased from Sigma (St. Louis, MO, USA). The serum was centrifuged at 14,000 g for 15 min to remove the lipids, and the supernatant was incubated for 15 min at 37°C before the assay. The half-lives of CATH2, TP5, and CbTP (10 µg/mL) were evaluated *in vitro* following incubation at 37°C in human serum at different time points. The samples were collected into prechilled tubes containing 1 mL of acidic acetone (hydrochloric acid/acetone/H_2_O, 1:40:5, by volume) and centrifuged at 2,000 ×g for 20 min at 4°C.

The obtained precipitates were dried in vacuo, after which the dried precipitates were dissolved in 0.5 mL of 1 M acetic acid. The peptide analysis was carried out according to the protocol for the study of TP5 using HPLC (26). The half-lives of the target peptides were calculated by a logarithm-linear regression analysis of the peptide concentrations.

### Animal Model

Sixty Balb/c female mice (6-8 weeks of age) were purchased from Charles River (Beijing, China) and maintained in specific-pathogen-free (SPF) conditions at room temperature. All the mice were allowed free access to feed and fresh water during the whole period. The experiment was conducted with the approval of the China Agricultural University Animal Care and Use Committee (Beijing, China).

The mice were randomly divided into the following eight groups (n=12): control group; cyclophosphamide (CTX) group, treated with CTX (80 mg/kg mouse weight; Sigma-Aldrich, St. Louis, MO, USA); CTX+CATH2 group, mice pretreated with CTX, followed by 10 mg/kg CATH2 treatment; CTX+TP5 group, mice pretreated with CTX, followed by 10 mg/kg TP5 treatment; and the CTX+ CbTP groups, mice pretreated with CTX, followed by 2.5, 5, 10 or 20 mg/kg CbTP treatment. For the first 3 days, CTX (80 mg/kg mouse weight) was intraperitoneally (i.p.) injected daily into the CTX, CTX+CATH2, CTX+TP5, and CTX+CbTP groups to establish the immunosuppressed animal model. An equal volume of sterile saline (HyClone) was administered i.p. to the control group each day. From days 4 to 10 (7 days), the parental peptide (CATH2 and TP5; 10 mg/kg mouse weight) or CbTP peptide (2.5, 5, 10 or 20 mg/kg mouse weight) were i.p. injected into mice (CTX+CATH2, CTX+TP5, and CTX+CbTP) each day. The control and CTX groups were given equal volume sterile saline only. Twenty-four hours after the last dose, the mice were euthanized by cervical dislocation and their tissues and blood were collected for analysis. The body weight of each mouse was recorded at the beginning and end of the experiment. Spleen and thymus indices were calculated in the following way: Index (mg/g) = weight of spleen or thymus/body weight.

### Flow Cytometry

The spleen was rinsed immediately with 40-μm mesh cell strainers filled with PBS to harvest cell suspensions after collection and grinding. The cells were stained using CD3-PerCP, CD4-APC and CD8-FITC for 30 min at 4°C for T lymphocyte measurements. Data were obtained from multicolor analysis using a Guava easyCyte 6-21 system and were then processed with Guavasoft (Millipore, Burlington, MA, USA).

### Serum Cytokine and Immunoglobulin (Ig) Measurements

Serum was collected from mice by centrifugation of whole blood samples at 1,000 × g for 20 min. Levels of TNF-α, IL-6, IL-1β, IgG, IgA, and IgM in the serum were determined by ELISA (eBioscience, San Diego, USA).

### Specific Binding of CbTP to TLR2

RAW264.7 cells analyzed for cytokine quantification in the culture supernatants were treated with the mouse IgG2a isotype control antibody or an anti-mouse TLR2 mAb (C9A12 Ab, InvivoGen, San Diego, CA, USA) for 1 h followed by the addition or no addition of 10 μg/mL CbTP peptide and further incubated for 24 h at 37°C. Levels of TNF-α, IL-6, and IL-1β in the culture supernatant were determined by ELISA (eBioscience).

Surface plasmon resonance (SPR) experiments were performed using a ProteOn XPR36 instrument (Bio-Rad, Hercules, CA, USA) with a ProteOn GLH sensor chip (Bio-Rad) at 25°C. The running buffer (PBS with 1% Tween 20) was continuously passed into reaction chamber. An SPR sensing chip with recombinant TLR2 (R&D Systems, Minneapolis, MN, USA) was immobilized by amino coupling to capture CbTP, as described in the manufacturer’s protocol. The binding affinity of CbTP to the TLR2-covered surface was examined in varying concentrations of the CbTP peptide (0, 1.25, 2.5, 5, and 10 μM). The running buffer was injected into the empty channels as a reference. To regenerate the chip surface at the end of each experiment, 10 mM Gly-HCl buffer (pH 2.5) was injected. Data analysis was conducted using ProteOn manager software (version 2.0). The binding curves were processed for the start injection alignment and baseline. The reference-subtracted sensorgram was then fitted to the curves labeling a homogeneous 1:1 model and protein surface data were grouped together to fit the association (*K*_a_) and dissociation (*K*_d_) rate constants. The equilibrium dissociation constant (*K*D) for the peptide-TLR2 interaction was calculated using the following equation:

KD=Kd/Ka

### Molecular Dynamics Simulation

The docking poses of CbTP-TLR2 were determined by RosettaDock (Version 3.5), and the missing hydrogen atoms in this system were added by Maestro at pH 7.0. The most favorable binding site with the lowest energy was selected for additional analysis. The best binding pose of CbTP with TLR2 was refined using molecular dynamics simulation with AMBER14 (27). The protein system was first treated with GAFF (27) and FF14SB (28) and then solvated with TIP3P (29) water molecules in a cubic box. The minimal distance between the edge of the box and the protein was 10 Å. The CbTP-TLR2 complex was neutralized by adding an appropriate number of Na^+^ and Cl^–^ atoms. The first 1,000 steps were used to minimize the system by the conjugate gradient algorithm, followed by heating gradually at a constant number, pressure, and temperature for 200 ps. Subsequently, a molecular dynamics simulation of 100 ps was performed under a constant number, volume, and temperature. The binding free energy for CbTP-TLR2 complex was calculated based on the molecular mechanics Poisson-Boltzmann accessible surface area (30). The particle-mesh Ewald method (31) was employed to address the long-range electrostatic interactions.

### Confocal Laser-Scanning Microscopy

RAW264.7 cells were treated with or without 10 μg/mL CbTP for 1 h at 37°C. Cells were then incubated (30 min, on ice) with anti-mouse monoclonal antibody TLR2 (1 μg/mL; eBioscience, San Diego, CA, USA) or isotype control IgG (eBioscience), washed, and stained (30 min, on ice) with a FITC-conjugated anti-mouse IgG antibody (Jackson Laboratories). After washing and adherence (15 min, room temperature) on cover glass-bottom confocal dishes, the cells were fixed with paraformaldehyde for 15 min at room temperature and mounted (Prolong Gold antifade reagent; Invitrogen). The cell nuclei were stained with DAPI (1:500 dilution in PBS) (Sigma-Aldrich). Cells were imaged with the Leica TCS SP5 confocal system (Leica Microsystems Ltd.). Images were processed and fluorescence was quantified with LAS AF software. In addition, to generate a fluorescence histogram profile, a line was drawn along the cell surface. Fluorescence intensities higher than 40 arbitrary units (isotype control staining) were considered clusters of TLR2 molecules.

### Western Blotting

Whole protein in the serum was collected (whole-protein extract kit, KeyGEN Biotech, Nanjing, China), and protein concentration was calculated using a bicinchoninic acid kit (Nanjing KeyGEN Biotech) as described in the manufacturer’s instructions. Protein supernatant was separated by 10% sodium dodecyl sulfate-polyacrylamide gel electrophoresis and transferred onto a nitrocellulose membrane. Next, the membranes were blocked with 5% defatted milk proteins in 0.05% TBST and probed with nuclear factor-κ-gene binding (NF-κB; p65), phosphor-NF-κB (p-NF-κB; p-p65), inhibitory subunit of NF-κB (IκB)-α, phosphor-IκB-α (p-IκB-α), inhibitor of NF-κB kinase (IKK)-β, phosphor-IKK-β (p-IKK-β), and β- actin-specific monoclonal antibodies (Santa Cruz, CA, USA) overnight at 4°C. Membrane sections were incubated with HRP-conjugated secondary antibody (Santa Cruz Biotechnology; 1:1000). ChemiDoc MP Imaging System (Bio-Rad, Hercules, CA, USA) was used to quantify the density of the specific proteins.

### Statistics

All experiments were carried out with at least three biological replicates. The data are presented as the means ± SD. Statistical comparisons were performed by Student’s t test and one-way ANOVA using GraphPad Prism 7 software (La Jolla, California). Significance was set as follows: not significant (NS), p > 0.05; *p ≤ 0.05; **p ≤ 0.01; ***p ≤ 0.001; and ****p ≤ 0.0001.

## Results

### Screening of Immunomodulatory Peptides

**Figure 1:**
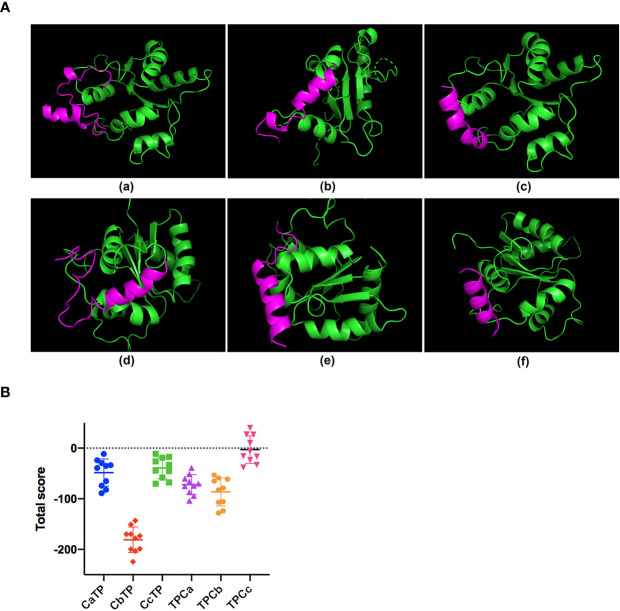
The binding ability of the hybrid peptides to TLR2. **(A)** The overall structure of the peptide-TLR2 complex. **(A-a)** TLR2-CaTP; **(A-b)** TLR2-CbTP; **(A-c)** TLR2-CcTP; **(A-d)** TLR2-TPCa; **(A-e)** TLR2-TPCb; **(A-f)** TLR2-TPCc. The green ribbons represent TLR2, and the red ribbons represent the hybrid peptide. **(B)** Energy plot of 10 of 100 decoy structures from the hybrid peptide-TLR2 docking study by flexpepdock. The data are shown as the mean ± standard deviation.

The sequences of the hybrid peptides (CaTP, CbTP, CcTP, TPCa, TPCb, and TPCc) and their parental peptides (CATH2 and TP5) are shown in **Table 1**. The 3D structure of the peptides was analyzed by PyMOL software. Molecular docking was performed to evaluate the interaction between the peptides and TLR2. As shown in **Figure 1**, CbTP had more favorable docking scores for TLR2 than the other hybrid peptides, and the total score was lower than -100.

**Figure 2:**
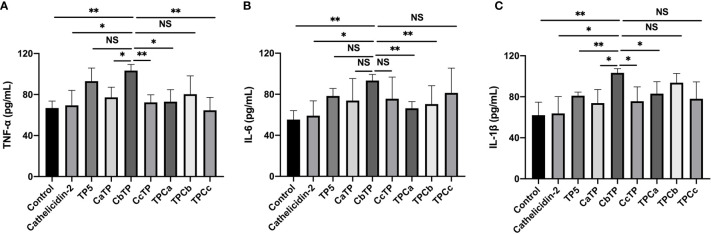
The *in vitro* immunomodulatory activity of the hybrid peptides in RAW264.7 cells. After administration of 10 μg/mL peptides for 12 h, the protein levels of TNF-α **(A)**, IL-6 **(B)** and IL-1β **(C)** in the RAW264.7 cell culture supernatant were quantified by ELISA. Statistical comparisons were performed by Student’s t test. The data are given as the mean ± standard deviation (n=5). NS: p > 0.05; *p ≤ 0.05; and **p ≤ 0.01.

**Figure 3:**
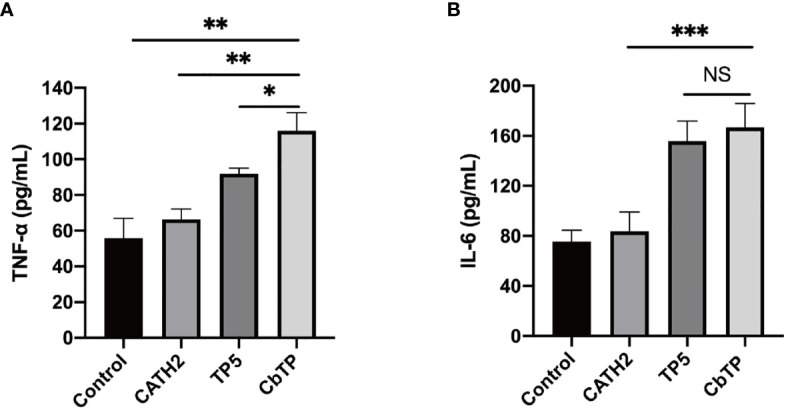
The *in vitro* immunomodulatory activity of CATH2, TP5, and CbTP in human THP-1 monocyte-macrophage cells. After administration of 10 μg/mL peptides for 12 h, the protein levels of TNF-α **(A)** and IL-6 **(B)** in the THP-1 cell culture supernatant were quantified by ELISA. Statistical comparisons were performed by Student’s t test. The data are given as the mean ± standard deviation (n=5). NS: p > 0.05, *p ≤ 0.05, **p ≤ 0.01, and ***p ≤ 0.001.

After the molecular docking assay, the immunomodulatory activities of these hybrid peptides were evaluated *in vitro*. As shown in **Figure 2**, the cell line RAW264.7 exhibited increases in the cytokine TNF-α, IL-6, and IL-1β (**Figure 2**) when treated with CbTP. In addition, the ability of CbTP to induce the expression of at least one of these immune cytokines is stronger than that of the parental peptides and other hybrid peptides designed in this study (**Figure 2**), which indicates that CbTP has stronger immunomodulatory potential. Therefore, CbTP, the most active peptide, was selected for further immunomodulatory experiments. The immunopotentiating activity of CbTP was also confirmed in human THP-1 monocyte-macrophage cells (**Figure 3**).

### Cytotoxicity on RAW264.7 Macrophage Cells

**Figure 4:**
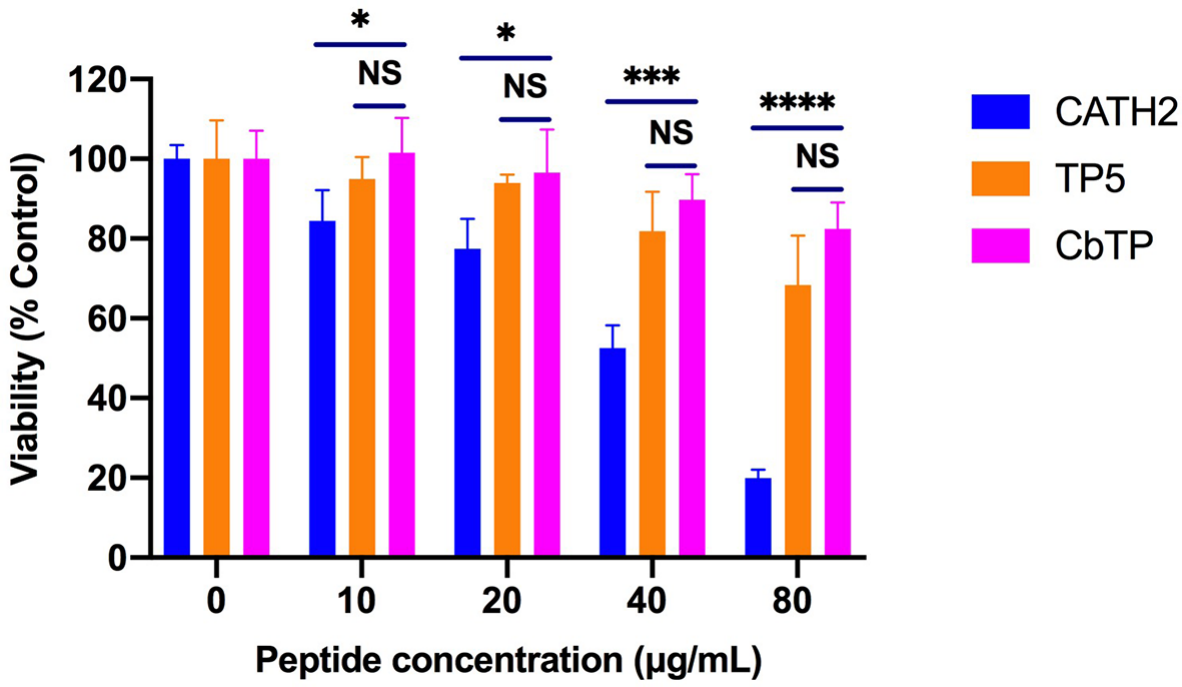
Cell viability of RAW264.7 macrophages. Cells were treated with various concentrations of peptides and incubated overnight at 37°C and 5% CO_2_. Cell proliferation rates were measured by microplate at 450 nm using CCK8 assay. Statistical comparisons were performed by Student’s t test. Data are given as the mean ± standard deviation (n=5). NS: p > 0.05, *p ≤ 0.05, ***p ≤ 0.001, and ****p ≤ 0.0001.

The cytotoxic activity of CbTP on RAW264.7 macrophage cells was evaluated by CCK-8 assay, and the results are shown in **Figure 4**. CbTP induced cytotoxicity in a dose-dependent manner, while even a high dose (80 µg/mL) of CbTP was nontoxic to RAW264.7 cells. In addition, the cytotoxicity of CbTP was lower than that of parental peptide CATH2.

### *Ex Vivo* Stability of CbTP in Human Serum

**Figure 5:**
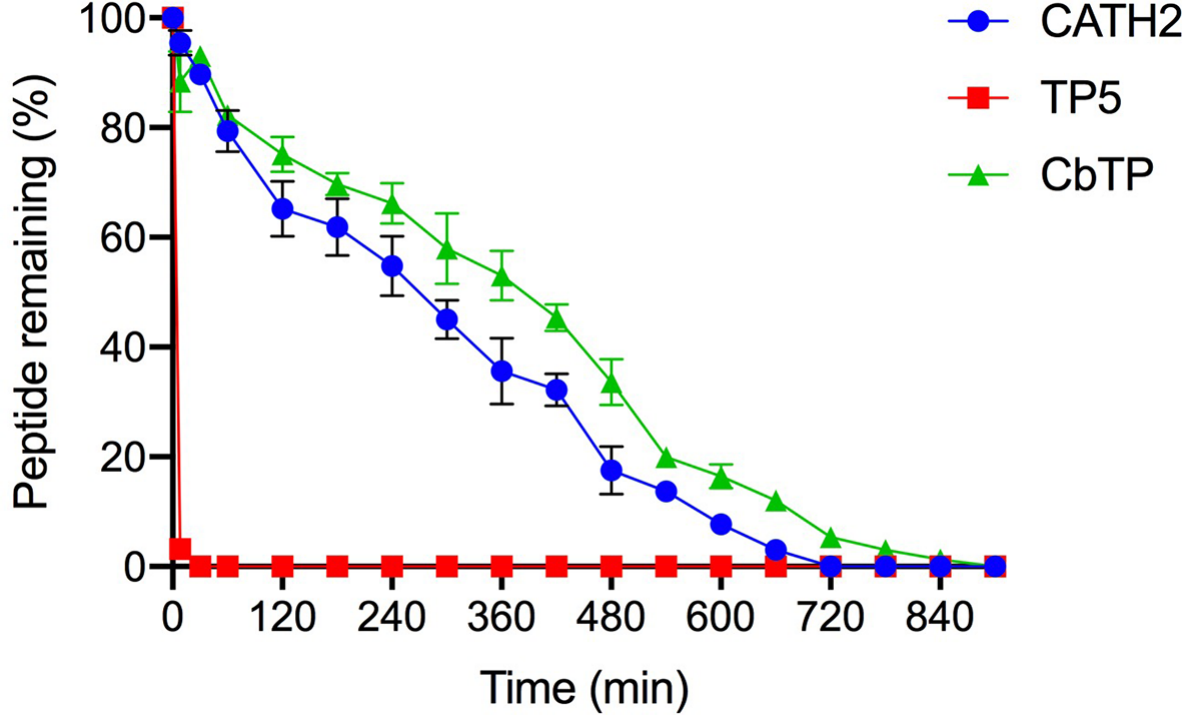
The mean human serum concentrations of CATH2, TP5, and CbTP over time. The concentration of each target peptide in serum *in vitro* at the selected time points was quantified by HPLC. The data are given as the mean ± standard deviation (n=5).

Human serum levels of peptides CATH2, TP5, and CbTP over time were analyzed and are shown in **Figure 5**. TP5 showed a very short half-life (t1/2) and its concentrations decreased nearly 95% after incubation in serum for 5 min. Compared with TP5, the newly designed peptide CbTP significantly prolonged the degradation profile (**Figure 5**) and half-life (360 min). However, there was no statistical significance between CbTP and CATH2 (**Figure 5** and **Table 2**).

**Table 2:** Half-life of CbTP in human serum.

Peptides	CATH2	TP5	CbTP
t_1/2_ (min)	302 ± 34.5^a^	3.2 ± 0.5^b^	353 ± 41.3^a^

Statistical comparisons were performed by ANOVA. The data are given as the mean ± standard deviation (n=5). Means with different superscript letters significantly differed (p ≤ 0.01).

### Effect of CbTP on Body Weight and Immune Organs

**Figure 6:**
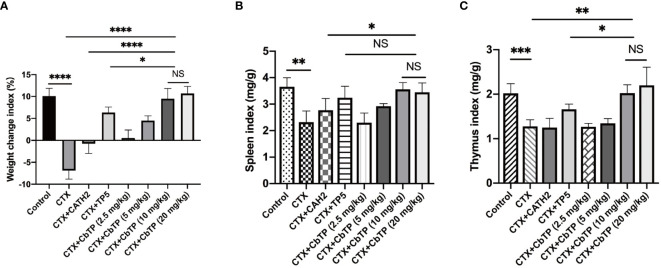
The effects of CbTP on body weight **(A)**, spleen index **(B)**, and thymus index **(C)**. The mice were randomly divided into eight groups (12 mice in each group): control group: physiological saline was injected intraperitoneally once daily; cyclophosphamide (CTX) group: CTX (80 mg/kg mouse weight) was injected intraperitoneally once daily for the first 3 days. From days 4 to 10, physiological saline was injected intraperitoneally into mice once daily; CTX + peptide (CATH2, TP5, or CbTP) group: CTX (80 mg/kg mouse weight) was injected intraperitoneally once daily for 3 consecutive days. From days 4 to 10, the parental peptide (CATH2 and TP5; 10 mg/kg mouse weight) or CbTP peptide (2.5, 5, 10 or 20 mg/kg mouse weight) was injected intraperitoneally into mice once daily. The body weight was recorded before and after the experiment. The spleen weights and thymus weights of the mice were examined after 10 days treatment. Statistical comparisons were performed by Student’s t test. The data are shown as the mean ± standard deviation (n=12). NS, p > 0.05; *p ≤ 0.05; **p ≤ 0.01; ***p ≤ 0.001; and ****p ≤ 0.0001.

In contrast to the CTX-induced group, which exhibited a significant decrease in body weight compared to the control group, mice in the CATH2-, TP5-, and CbTP-treated groups exhibited less loss in body weight. In addition, body weight in the CbTP-treated group was significantly higher than that in the parental peptide (CATH2 and TP5) treated groups at the same concentration (**Figure 6A**).

We found that the mice in the CTX group had significantly lower spleen (**Figure 6B**) and thymus (**Figure 6C**) index values than the mice in the control group. However, the administration of CbTP remarkably improved the spleen and thymus indices, and the thymus index value in the CbTP-treated group were significantly higher than those in TP5-treated and C-treated groups at the same concentration.

### Effects of CbTP on Serum Cytokines and Immunoglobulin Levels

**Figure 7:**
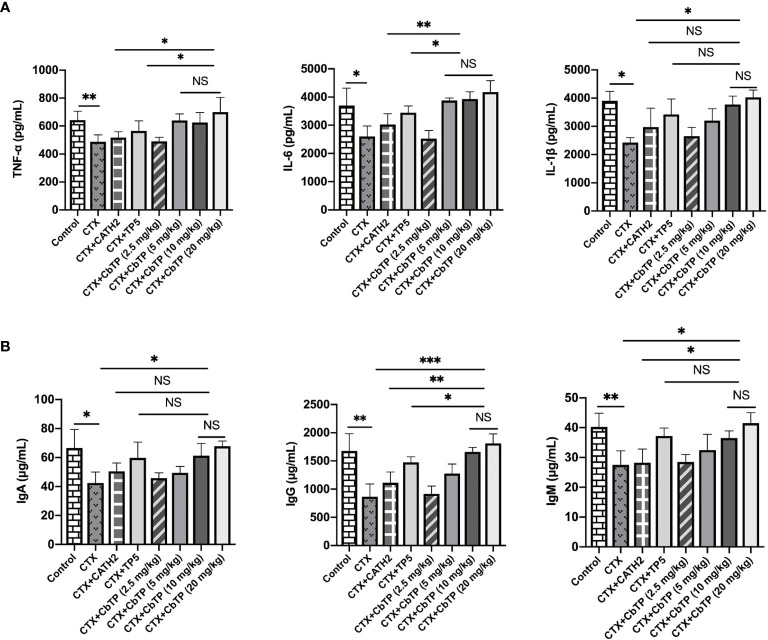
Effects of CbTP on serum cytokines and immunoglobulin levels. **(A)** Effects of CATH2 (10 mg/kg mouse weight), TP5 (10 mg/kg mouse weight), or CbTP (2.5, 5, 10 or 20 mg/kg mouse weight) on TNF-α, IL-6, and IL-1β levels in mice immunosuppressed by cyclophosphamide (CTX). ELISA quantification of TNF-α, IL-6, and IL-1β levels in the serum of mice from different groups. **(B)** Effects of CATH2 (10 mg/kg mouse weight), TP5 (10 mg/kg mouse weight), or CbTP (2.5, 5, 10 or 20 mg/kg mouse weight) on IgA, IgG, and IgM levels in mice immunosuppressed by CTX. Levels of IgA, IgG, and IgM in the serum of mice from different groups were determined through ELISA. Statistical comparisons were performed by Student’s t test. The data are shown as the mean ± standard deviation (n=12). NS: p > 0.05, *p ≤ 0.05, **p ≤ 0.01, and ***p ≤ 0.001.

To evaluate the protective effects of CbTP against immunosuppression in CTX-treated mice, TNF-α, IL-6, and IL-1β in the serum of mice were measured. As shown in **Figure 7A**, CTX treatment resulted in significant reductions in TNF-α, IL-6, and IL-1β levels. The administration of CbTP caused a marked increase in all these cytokine levels in the serum of mice. Notably, treatment with CbTP (10 mg/kg mouse weight) resulted in higher TNF-α and IL-6 levels than in the CTX + CATH2 and CTX + TP5 groups (**Figure 7A**).

Furthermore, serum levels of immunoglobulins, including IgM, IgG and IgA, were severely decreased after CTX treatment (**Figure 7B**). CbTP reversed the declines in IgA, IgG and IgM caused by CTX stimulation. Compared with the CATH2- or TP5-treated group, CbTP had significantly higher IgG levels at the same concentration. The IgM level in mice treated with CbTP (10 mg/kg mouse weight) were similar to those in mice treated with TP5, which was significantly higher than those for the mice in the CATH2-treated group (**Figure 7B**).

In summary, CbTP peptide dose-dependently increased the levels of these cytokines and immunoglobulins at concentrations below 10 mg/kg. However, the cytokine and immunoglobulin levels in CTX+10 mg/kg CbTP group and CTX+20 mg/kg CbTP group were not statistically different (**Figure 7**). Therefore, in the follow-up study of CbTP in this article, a concentration of 10 mg/kg was adopted.

### Effects of CbTP on T Cells in Mouse Splenocytes

**Figure 8:**
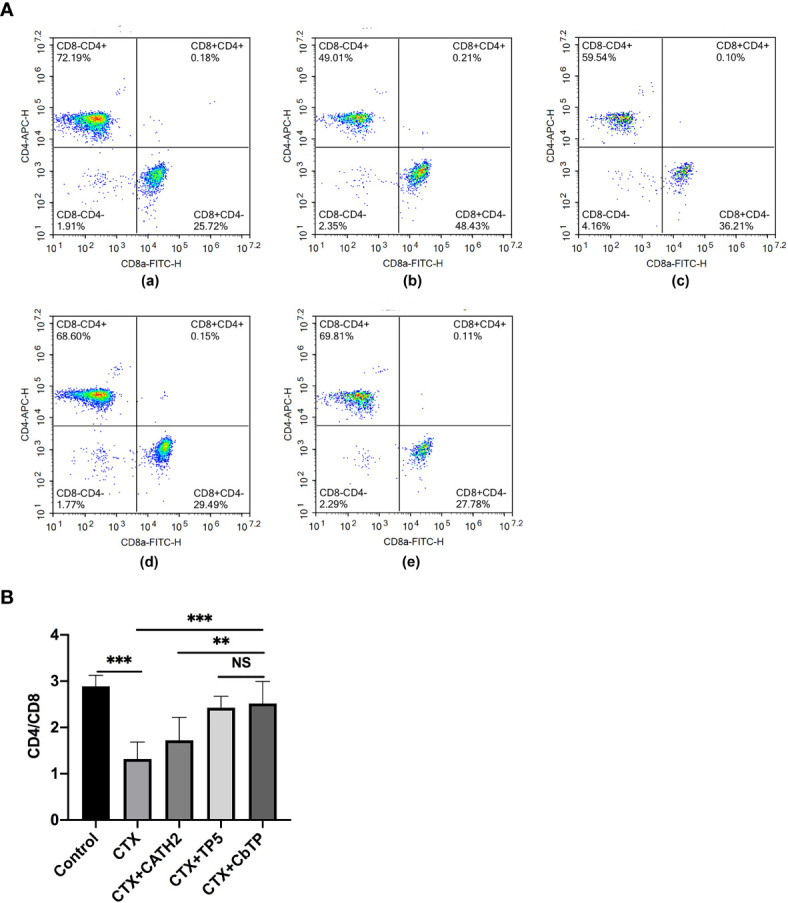
Effects of CbTP on T lymphocyte subpopulations in splenocytes. The spleen was rinsed immediately with 40-μm mesh cell strainers filled with PBS to harvest cell suspensions after collection and grinding. The cells were stained using CD3-PerCP, CD4-APC and CD8-FITC for 30 min at 4°C for T lymphocyte measurements. **(A)** The percentage of different T cell subsets was analyzed by flow cytometry. **(A-a)** Control, **(A-b)** CTX, **(A-c)** CTX + CATH2, **(A-d)** CTX + TP5, **(A-e)** CTX + CbTP. Bivariate plots are shown as representative assessments, which were quantified and plotted as the ratio of CD4^+^/CD8^+^ cells in part **(B)**. Statistical comparisons were performed by Student’s t test. The data are shown as the mean ± standard deviation (n=12). NS, p > 0.05; **p ≤ 0.01; and ***p ≤ 0.001.

The CD4^+^/CD8^+^ T lymphocyte ratio in the spleen was measured by the flow cytometry assay, and the results are shown in **Figure 8**. The CD4^+^/CD8^+^ T lymphocyte ratio decreased significantly in the CTX group compared with that of the control group. Treatments with CbTP significantly increased the proportions of CD4^+^ and CD8^+^ T lymphocytes. In addition, the increasing CD4^+^/CD8^+^ T lymphocyte ratio in the CbTP-treated group was significantly higher than that in the CATH2-treated group and similar to that in the TP5-treated group.

### CbTP Interacts Directly With TLR2 to Activate TLR2 Signaling

**Figure 9:**
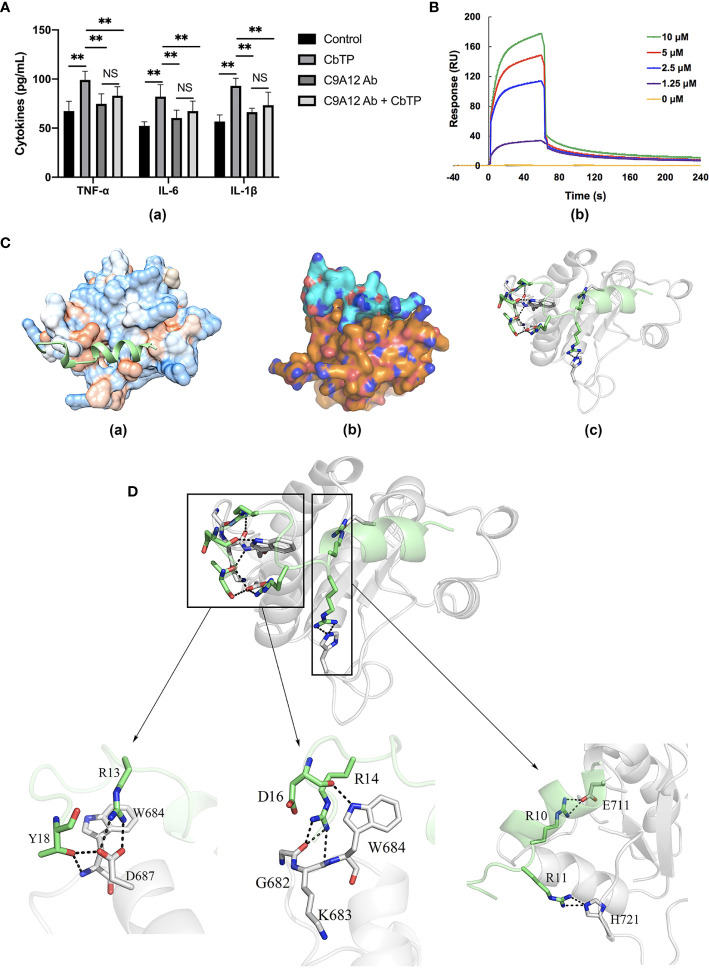
CbTP interacts directly with TLR2 to activate TLR2 signaling. **(A)** RAW264.7 cells analyzed for cytokine quantification in the culture supernatants were treated with PBS or an anti-mouse TLR2 mAb (C9A12 Ab) for 1 h followed by the addition or no addition of 10 μg/mL CbTP peptide and further incubated for 24 h at 37°C. Levels of TNF-α, IL-6, and IL-1β in the culture supernatant were determined by ELISA. NS: p > 0.05 and **p ≤ 0.01. **(B)** Specific binding of CbTP to TLR2. TLR2 was immobilized on a sensor chip, and CbTP binding was analyzed by surface plasmon resonance. **(C-c)** Binding modes between TLR2 and CbTP. Modeled crystal structure of CbTP bound in the hydrophobic region of TLR2. Best scoring tertiary structure model generated from docking procedure depicted as **(C-a)** Hydrophobic surface (orange: hydrophobic, blue: hydrophilic). The peptide CbTP is shown in green. **(C-b)** Electronic binding surface of the TLR2/CbTP complex (orange: TLR2, cyan: CbTP). **(C-c)** Amino acids forming the binding interface (TLR2: gray; CbTP: green). **(D)** Illustration of key residues of CbTP in the TLR2 interface. Close-up views of the CbTP-occupied sites in TLR2 are displayed. The light gray ribbons represent TLR2 and the hydrogen bonds are shown as black dashes.

RAW264.7 cells were incubated with PBS or TLR2 mAb for 1 h followed by the addition or no addition of 10 μg/mL CbTP peptide and further incubated for 24 h at 37°C. Levels of TNF-α, IL-6, and IL-1β in the culture supernatant were detected by ELISA. As shown in **Figure 9A**, RAW264.7 cells incubated with CbTP showed a significantly increased production of TNF-α, IL-6, and IL-1β compared with the control. However, pretreatment with TLR2 mAb significantly inhibited the TNF-α, IL-6, and IL-1β production induced by CbTP, implicating that the CbTP-TLR2 interaction is required for CbTP immunomodulatory signaling activation.

To analyze the interaction of CbTP and TLR2, an SPR assay was used to evaluate the binding kinetics of ligand-receptor interactions in detail (**Figure 9B**). Five different concentrations of CbTP (0, 1.25, 2.5, 5, and 10 μM) were passed over immobilized TLR2. The results indicated that CbTP binding to the chip-bound TLR2 protein exhibited a dose-dependent increase. The K*_a_* and K*_d_* values for CbTP binding to TLR2 were 3.61 × 10^8^ s^–1^ and 8.58×10-2 M^–1^s^–1^, respectively, and the K*_D_* was 2.38×10^-4^ μM (**Figure 9B**).

To investigate the detailed molecular interaction of CbTP and TLR2, we performed molecular dynamics simulations (**Figures 9C, D**). The hydrophilic and hydrophobic surface and electrostatic interaction analyses of TLR2 and CbTP showed excellent hydrophilic and hydrophobic matching and molecular charge similarity, indicating that there is a good binding phase between the two (**Figure 9C**). A total of 300 snapshots were observed from the last stable 60 ns of the molecular dynamics simulations for the CbTP-TLR2 complex. As shown in **Tables 3** and **4**, key parameters of the interaction between TLR2 and CbTP were analyzed, including the number of hydrogen bonds and salt bridges, the interaction surface area, the binding free energy, and the distance between the binding residues of CbTP and TLR2. The binding free energy (-975.6 kJ/mol) was correlated to the binding affinity calculated for the CbTP-TLR2 interaction pair (**Table 3**). The interface of TLR2 that binds CbTP was 324 Å^2^, and the interaction between CbTP and TLR2 was primarily mediated by hydrogen bonds and salt bridges (**Tables 3**, **4** and **Figure 9D**).

**Table 3:** Key interaction parameters between CbTP and TLR2.

Interaction pair	Number of hydrogen bonds	Number of salt-bridges	Interaction Surface (Å2)	Binding free energy (kj/mol)
TLR2…CbTP	15	8	324	-975.6

**Table 4:** The distance and salt bridges of the binding residues between CbTP and TLR2.

Interaction Pair TLR2…CbTP	Distance (Å)	Number of salt bridges
H_721_…R_11_	2.80	2
E_711_…R_10_	2.81	2
D_687_…R_13_	2.73	2
D_687_…Y_18_	3.21	2
W_684_…Y_18_	3.13	0
W_684_…D_16_	2.81	0
K_683_…R_14_	3.42	0

**Figure 10:**
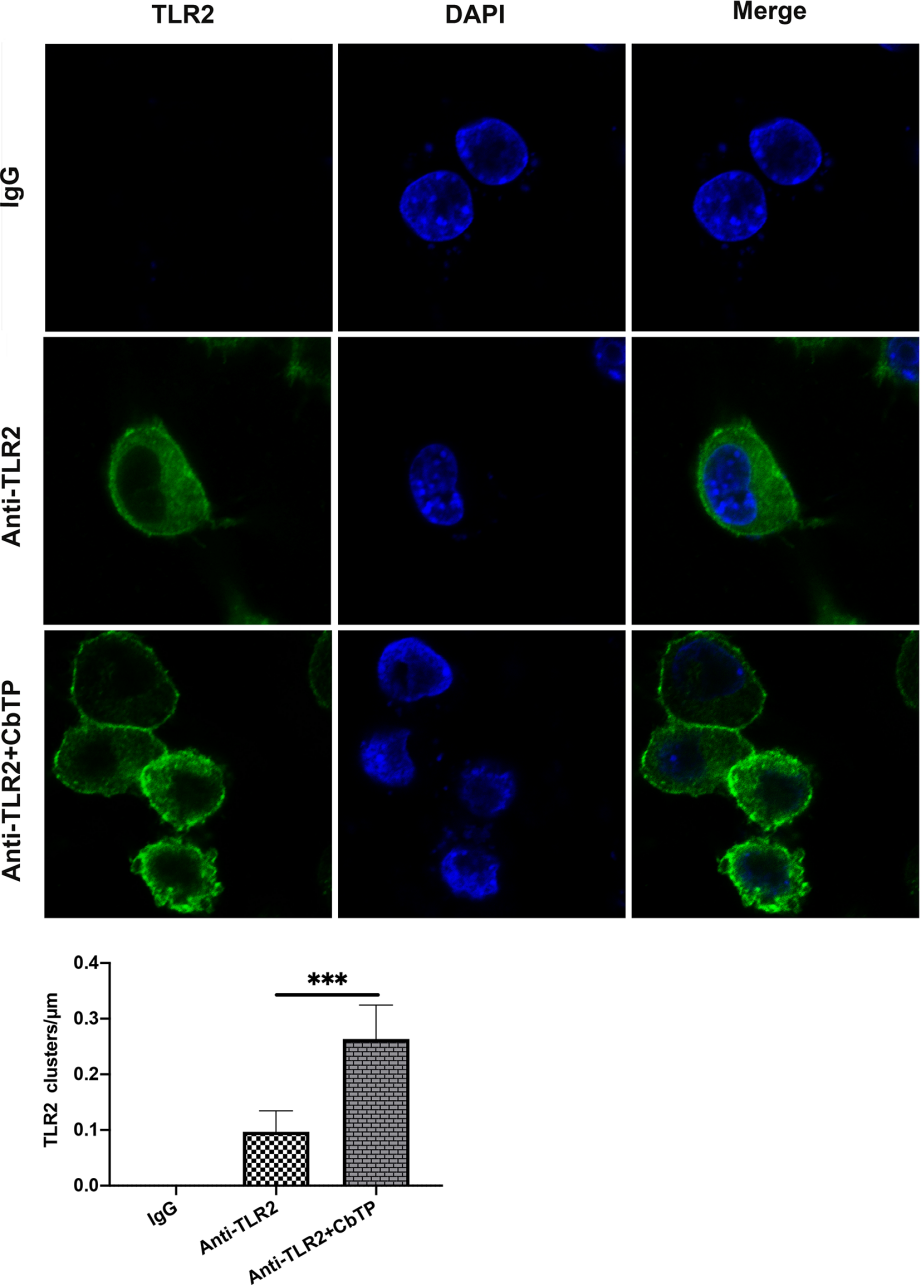
CbTP enhances cell surface TLR2 cluster formation. RAW264.7 cells were treated with or without 10 μg/mL CbTP for 1 h at 37°C. Cells were then incubated with anti-mouse monoclonal antibody TLR2 (1 μg/mL) or isotype control IgG, washed, and stained with a FITC-conjugated anti-mouse IgG antibody. After washing and adherence to cover glass-bottom confocal dishes, the cells were fixed with paraformaldehyde for 15 min at room temperature and mounted. The cell nuclei were stained with DAPI. Cells were imaged with the Leica TCS SP5 confocal system. Images were processed and fluorescence was quantified with LAS AF software. In addition, to generate a fluorescence histogram profile, a line was drawn along the cell surface. Fluorescence intensities higher than 40 arbitrary units (isotype control staining) were considered clusters of TLR2 molecules. Statistical comparisons were performed by Student’s t test. The mean values ± standard deviation of 5 independent experiments were used to express the data. ***p ≤ 0.001.

To explore the mechanism underlying the capacity of CbTP to modulate TLR2 activity, we first examined CbTP-induced cell surface TLR2 cluster formation because TLR clustering is believed to be critical to signaling (32, 33). In the absence of CbTP, TLR2 showed an even distribution on the monocyte cell surface with very few clusters per micrometer (**Figure 10**). In contrast, the addition of peptide CbTP to the cell culture resulted in a marked increase in the number of major TLR2 clusters per micrometer on the cell surface (**Figure 10**).

**Figure 11:**
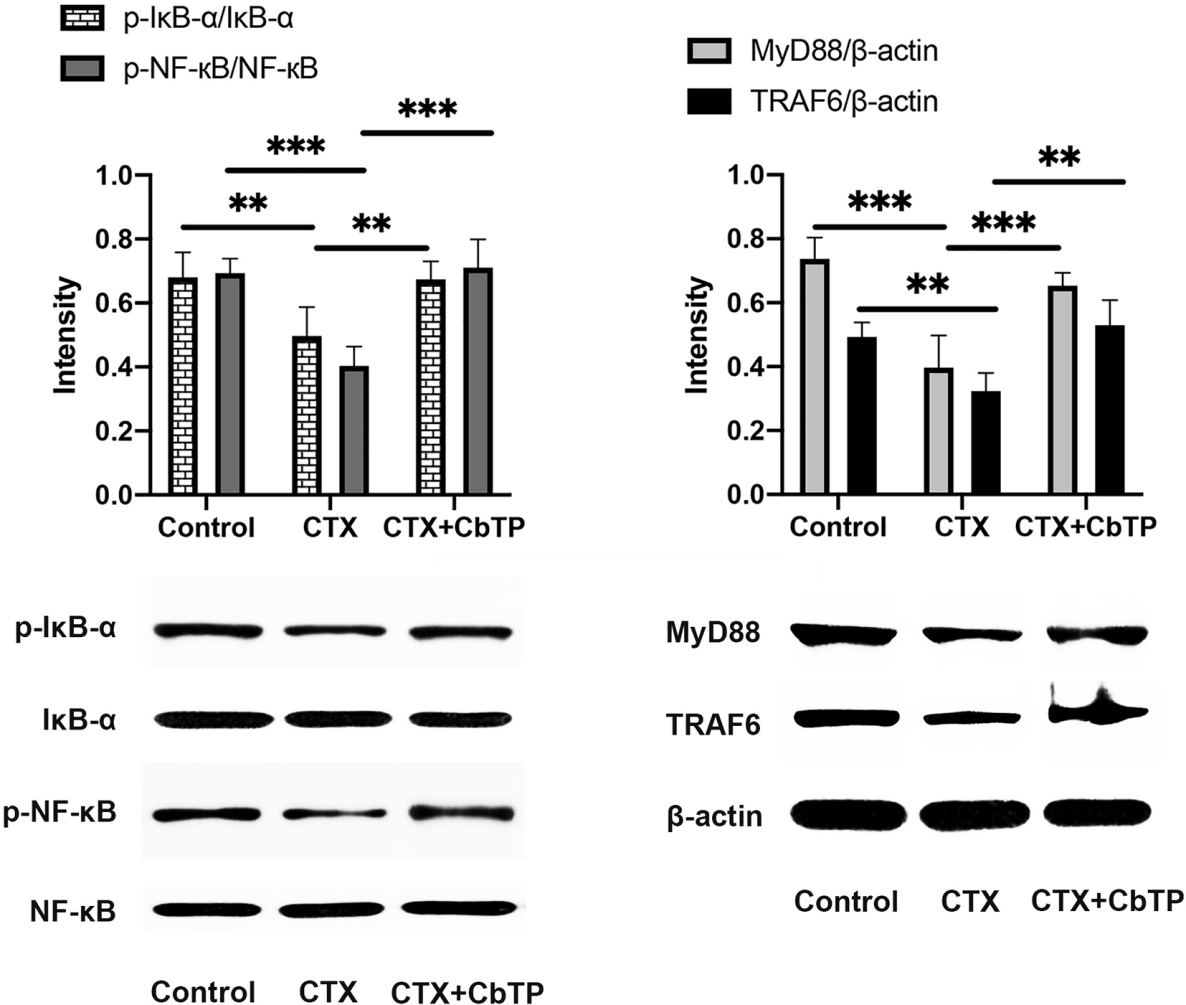
CbTP promotes the MyD88-NF-κB signaling pathway in the serum of mice. Total and phosphorylated protein levels of MyD88, TRAF6, IκB-α, NF-κB and β-actin in the serum, as depicted by western blotting. Statistical comparisons were performed by Student’s t test. Data are given as the mean ± standard deviation (n = 5). **p ≤ 0.01; ***p ≤ 0.001.

Next, to examine the mechanism underlying the immunomodulatory activity of CbTP, we studied the downstream signaling pathway of TLR2 (**Figure 11**). We found that CTX decreased the expression levels of MyD88 and TRAF6 and the phosphorylation of IкB-α and NF-кB. However, CbTP treatment significantly suppressed the decrease in the expression levels of MyD88 and TRAF6 and the phosphorylation of IкB-α and NF-кB mediated by CTX (**Figure 11**). These data indicate that CbTP could exert its immunomodulatory activity by activating the MyD88-NF-кB signaling pathway.

## Discussion

Immunosuppression is a state of temporary or permanent dysfunction of the immune response resulting from insults to the immune system and leading to increased susceptibility to disease (3). Although there are already some immunopotentiating agents that can be used to treat the clinical signs of immunosuppression and in some cases can induce disease remission, these immunopotentiators are not satisfactory from the perspective of immunological safety and effectiveness (34). Thus, it is necessary to explore novel safe and efficient alternatives for immunosuppression therapy.

Previously, it was reported that some peptides elicit certain immunopotentiating activities, such as TP5 (20, 21) and CATH2 (15–18). However, their potential cytotoxicity (15), relatively limited immunoregulatory activity (15–18), and weak physiological stability (20, 21) greatly reduce their clinical development. Peptide hybridization is an effective strategy for the design of novel functional peptides because hybrid peptides could combine the advantages of different native peptides. In this study, we designed six hybrid peptides by combining TP5 with the active fragments of CATH2 to improve the immunoregulatory activity and physiological stability and reduce the cytotoxicity of the parental peptides.

Given that TLR2 is a very important signal transduction protein in the immune regulation process (4–6, 9), and there are signs that both TP5 and CATH2 can regulate the signal pathway mediated by TLR protein (14, 15, 19), we therefore regard TLR2 as a major target protein to evaluate and screen hybrid peptides with excellent immunomodulatory potential. Initially, the binding modes of the hybrid peptides were simply and effectively screened by molecular docking. Among six newly designed peptides, CbTP had the most favorable docking score for TLR2. Additionally, the immunomodulatory activities of the hybrid peptides were assessed by in RAW264.7 cells. Cytokines are pleiotropic signaling molecules that not only mediate cell-to-cell communication but also play important roles in the innate and adaptive immune systems (35, 36). Immune cells release large amounts of cytokines, such as TNF-α, IL-6, and IL-1β, into circulation to induce and/or modulate immune responses and respond to perturbations (35–37). We demonstrated that CbTP caused a more significant increase in the secretion of TNF-α, IL-6, and IL-1β in comparison with the other peptides tested. In addition, the immunopotentiating activity of CbTP was confirmed in human THP-1 monocyte-macrophage cells. Therefore, CbTP was selected for further immunomodulatory experiments.

The peptides designed in this study aimed to obtain a peptide with more potent immunoenhancing activity and physiological stability but reduced cytotoxicity. Thus, we evaluated the cytotoxic activity of the hybrid peptide by CCK-8 assay. CbTP exhibited lower cytotoxicity than the parental peptides and showed very little cytotoxic activity at relatively high concentrations, which is a favorable feature for the therapeutic use of this peptide.

TP5, a parental peptide of the hybrid peptide CbTP, is an effective immunomodulatory agent for immunodeficiency-related diseases that has been used clinically for decades. However, its application is greatly limited by its extremely short half-life *in vivo*, resulting in repeated injection and poor patient compliance (20–22). In view of the half-life of peptides significantly affecting the dosage and therapy, it becomes an important task to prevent rapid degradation and prolong the half-life of such drugs. In this study, the half-life of CbTP in plasma was prolonged to 300 min, which was significantly longer than that of CATH2 or TP5 (less than 5 min) in plasma.

CTX, an alkylating agent, is the most commonly used immunosuppressant (38–40). It can greatly interfere with the differentiation and proliferation of T and B cells, kill immune cells, and decrease cellular and humoral immune responses (38–40). In this study, we established a CTX-induced mouse model to investigate the immune potentiating activity of CbTP and its potential as a new therapeutic to replace or supplement traditional immune drugs. As anticipated, CTX stimulation of mice resulted in a significant decrease in the body weight and spleen and thymus index values. Compared to the CTX group, the body weight and thymus index values in the CTX + C, CTX + TP5, and CTX + CbTP groups were significantly elevated, especially in the CTX + CbTP group, demonstrating the ability of CbTP to ameliorate CTX-induced damage to the body and immune organs.

T lymphocytes are the key players of the adaptive immune response, which both coordinate other immune cells and destroy malignant and virus-infected cells (41–44). When the immune system is suppressed, the organism is more susceptible to infection due to the decrease in the CD4^+^/CD8^+^ ratio (41–44). Consistent with a previous study (45), CTX remarkably reduced the proportions of CD4^+^ and CD8^+^ T lymphocytes. However, treatment with peptides significantly increased the CD4^+^/CD8^+^ ratio, and the increasing level in the CbTP and TP5-treated group was significantly higher than that in the CATH2-treated group, which indicated that CbTP can efficiently regulate immunity by adjusting the ratio of T lymphocyte subsets.

As immune cytokines are among the most crucial elements for the activation and enhancement of immune properties, we tested the changes in several representative immune cytokines in the present study (37). We demonstrated that treatment with CbTP significantly improved the levels of TNF-α, IL-6, and IL-1β. Despite the obvious enhancement effects of TP5 and CATH2 on CTX-induced immunosuppression, the immunomodulatory activities of the parental peptides were weaker than that of the hybrid peptide CbTP.

It has been proposed that immunoglobulins could act as potentiators of the immune response in tissues by means of antigen uptake to dendritic cells (46, 47). In addition, immunoglobulins can contribute directly to the immune response, including opsonization of antigens for destruction and fixation of complement and neutralization of toxins and viruses (46–48). In this study, stimulation by CTX led to a typical decrease in the levels of serum IgA, IgM, and IgG. Comparatively, CTX + CbTP treatment effectively upregulated the levels of these immunoglobulins. Furthermore, it is worth noting that CbTP-treated mice had significantly higher total serum IgA, IgG, and IgM levels than mice in the CATH2-treated groups. In addition to these general immune parameters, antigen-specific immune response experiments are also needed in future studies to further demonstrate the effectiveness of CbTP.

Taken together, the above data demonstrated that the newly designed hybrid peptide, CbTP, has significantly lower cytotoxicity and higher immunoregulatory activity and physiological stability than its parental peptides. These findings strongly support the therapeutic potential of CbTP against immunosuppression and hypoimmunity. To identify the underlying immunomodulatory mechanisms, a comprehensive and detailed analysis was conducted.

First, to investigate whether CbTP exhibited immunomodulatory properties by binding to TLR2 as proposed above, ELISA assays were performed. Treatment with CbTP significantly increased the levels of TNF-α, IL-6, and IL-1β, but pretreatment with TLR2 mAb significantly inhibited the cytokine production induced by CbTP, suggesting that CbTP exhibits immunomodulatory effects through interaction with the TLR2 signal transduction receptor. The SPR assay further confirmed that CbTP can efficiently bind to the TLR2 protein. Molecular dynamic simulations were then employed to investigate the precise binding sites and detailed mode of CbTP binding to TLR2. It has been demonstrated that CbTP can establish sustainable interactions with TLR2 by hydrogen bonds, which play a crucial role in initiating immune responses (49). According to some studies, low binding energy is an important characteristic that imparts high molecular dynamic affinity (50, 51). Hence, CbTP has great immunopotentiating potential because it has fairly low binding energy, therefore allowing for of efficient stimulation to TLR2 to further activate the immune response. Additionally, the current results showed that CbTP possessed a large interaction surface and many interacting amino acid-base pairs and salt bridges with the TLR2 receptor, which may also contribute to TLR2-stimulating activity and thus activate downstream immunoregulation-related signaling pathways.

TLR2 was reported to form clusters and to engage its ligand on the cell membrane where signaling was initiated (32, 33, 52). Therefore, in this study, we examined cell surface TLR2 cluster formation induced by CbTP. Confocal laser scanning microscopy provides clear experimental evidence that CbTP could significantly promote the formation of TLR2 clusters on the cell surface, which indicated that CbTP enhanced immune function by forming TLR2 clusters. Taken together, CbTP effectively improves immune ability by stimulating TLR2 and promoting the formation of TLR2 clusters, which might constitute an alternative strategy for the identification of new immunopotentiating drugs whose mechanisms of action involve TLR2.

A voluminous body of literature has revealed important roles for the NF-κB pathway in regulating different aspects of immune functions (53, 54). MyD88-dependent signaling is a principal pathway that regulates cytokines, such as IL-1β, IL-6, and TNF-α, and cells that participate in the immunoregulation process (55). MyD88 is utilized by TLR2, recruits TRAF6, and then activates NF-κB signaling (54). Given this background, we decided to assess the changes in key factors involved in this pathway. The results indicated that CbTP significantly increased the expression of MyD88 and TRAF6 and the phosphorylation of IκB-α and NF-κB, indicating that CbTP could successfully activate the MyD88-NF-κB pathway, eventually contributing to the immunoregulation of cytokine expression.

## Conclusion

A novel approach for the design of new immunoregulatory peptides through the hybridization of different native peptides that have immunoregulatory activity was developed to design the hybrid peptide CbTP. CbTP possessed significantly lower cytotoxicity and higher immunopotentiating activity and physiological stability than its parental peptides CATH2 and TP5. The immunomodulatory effects of the newly designed hybrid peptide CbTP were confirmed in CTX-immunosuppressed mouse model. CbTP successfully inhibited immunosuppression and weight loss, increased immune organ indices, and improved CD4^+^/CD8^+^ T lymphocyte subsets. In addition, CbTP significantly increased the production of the cytokine TNF-α and IL-6 and the immunoglobulins IgA, IgM, and IgG. The immunoenhancing effects of CbTP were attributed to its TLR2-binding activity, promoting the formation of the TLR2 cluster, the activation of the TLR2 receptor, and activation of the downstream MyD88-NF-κB signaling pathway. Thus, peptide hybridization is a promising approach for the design and development of new immunoregulatory peptides with enhanced bioavailability and limited adverse effects. The immunoregulatory potential of these peptides can be exploited in technological and clinical applications, such as food additives, healthcare formulas, or therapeutic immunoregulatory drugs.

## Data Availability Statement

The original contributions presented in the study are included in the article/supplementary material. Further inquiries can be directed to the corresponding author.

## Ethics Statement

The animal study was reviewed and approved by China Agricultural University Animal Care and Use Committee (Beijing, China).

## Author Contributions

XW, LZ, RZ, RW, JP, and YH conceived and designed the experiments. XW, LZ, DS, BA, HG MZ, QC, and YT performed experiments and evaluated the data. XW prepared the manuscript and JP revised the manuscript. All authors contributed to the article and approved the submitted version.

## Funding

This work was funded by the National Key Research and Development Program of China (grant number 2018YFD0500600) and National Natural Science Foundation of China (grant numbers 31572442 and 31272476).

## Conflict of Interest

The authors declare that the research was conducted in the absence of any commercial or financial relationships that could be construed as a potential conflict of interest.

## References

[1] Toskala E. Immunology. Int Forum Allergy Rhinol (2014) 4(Suppl 2):S21–7. 10.1002/alr.21380 25182350

[2] Hooper LV Littman DR Macpherson AJ. Interactions Between the Microbiota and the Immune System. Science (2012) 336(6086):1268–73. 10.1126/science.1223490 PMC4420145 22674334

[3] Goldszmid RS Dzutsev A Trinchieri G. Host Immune Response to Infection and Cancer: Unexpected Commonalities. Cell Host Microbe (2014) 15(3):295–305. 10.1016/j.chom.2014.02.003 24629336 PMC3996827

[4] Delarosa O Dalemans W Lombardo E. Toll-Like Receptors as Modulators of Mesenchymal Stem Cells. Front Immunol (2012) 3:182. 10.3389/fimmu.2012.00182 22783256 PMC3387651

[5] Akira S Uematsu S Takeuchi O. Pathogen Recognition and Innate Immunity. Cell (2006) 124(4):783–801. 10.1016/j.cell.2006.02.015 16497588

[6] Oliveira-Nascimento L Massari P Wetzler LM. The Role of TLR2 in Infection and Immunity. Front Immunol (2012) 3:79. 10.3389/fimmu.2012.00079 22566960 PMC3342043

[7] Fereig RM Abdelbaky HH Kuroda Y Nishikawa Y. Critical Role of TLR2 in Triggering Protective Immunity With Cyclophilin Entrapped in Oligomannose-Coated Liposomes Against Neospora Caninum Infection in Mice. Vaccine (2019) 37(7):937–44. 10.1016/j.vaccine.2019.01.005 30660401

[8] Zhang LL Wei XB Zhang RJ Petitte J Si DY Li ZX. Design and Development of a Novel Peptide for Treating Intestinal Inflammation. Front Immunol (2019) 10:1841. 10.3389/fimmu.2019.01841 31447849 PMC6691347

[9] Brzezinska-Blaszczyk E Wierzbicki M. [Mast Cell Toll-Like Receptors (Tlrs)]. Postepy Hig Med Dosw (Online) (2010) 64:11–21. 20093719

[10] Goldstein G Scheid MP Boyse EA Schlesinger DH Vanwauwe J. Synthetic Pentapeptide With Biological-Activity Characteristic of the Thymic Hormone Thymopoietin. Science (1979) 204(4399):1309–10. 10.1126/science.451537 451537

[11] Singh VK Biswas S Mathur KB Haq W Garg SK Agarwal SS. Thymopentin and Splenopentin as Immunomodulators - Current Status. Immunol Res (1998) 17(3):345–68. 10.1007/Bf02786456 9638477

[12] Mascart-Lemone F Huygen K Clumeck N Brenez D Bolla K Duchateau J. Stimulation of Cellular Function by Thymopentin (TP-5) in Three AIDS Patients. Lancet (1983) 2(8352):735–6. 10.1016/s0140-6736(83)92271-7 6193382

[13] Sundal E Bertelletti D. Thymopentin Treatment of Rheumatoid Arthritis. Arzneimittelforschung (1994) 44(10):1145–9. 7818590

[14] Li J Cheng Y Zhang X Zheng L Han Z Li P. The in Vivo Immunomodulatory and Synergistic Anti-Tumor Activity of Thymosin Alpha1-Thymopentin Fusion Peptide and Its Binding to TLR2. Cancer Lett (2013) 337(2):237–47. 10.1016/j.canlet.2013.05.006 23684552

[15] van Dijk A van Eldik M Veldhuizen EJ Tjeerdsma-van Bokhoven HL de Zoete MR Bikker FJ. Immunomodulatory and Anti-Inflammatory Activities of Chicken Cathelicidin-2 Derived Peptides. PLoS One (2016) 11(2):e0147919. 10.1371/journal.pone.0147919 26848845 PMC4743981

[16] van Dijk A Molhoek EM Veldhuizen EJ Bokhoven JL Wagendorp E Bikker F. Identification of Chicken Cathelicidin-2 Core Elements Involved in Antibacterial and Immunomodulatory Activities. Mol Immunol (2009) 46(13):2465–73. 10.1016/j.molimm.2009.05.019 19524300

[17] Molhoek EM van Dijk A Veldhuizen EJ Dijk-Knijnenburg H Mars-Groenendijk RH Boele LC. Chicken Cathelicidin-2-Derived Peptides With Enhanced Immunomodulatory and Antibacterial Activities Against Biological Warfare Agents. Int J Antimicrob Agents (2010) 36(3):271–4. 10.1016/j.ijantimicag.2010.06.001 20630709

[18] Scheenstra MR van den Belt M Tjeerdsma-van Bokhoven JLM Schneider VAF Ordonez SR van Dijk A. Cathelicidins PMAP-36, LL-37 and CATH-2 are Similar Peptides With Different Modes of Action. Sci Rep (2019) 9(1):4780. 10.1038/s41598-019-41246-6 30886247 PMC6423055

[19] Coorens M Scheenstra MR Veldhuizen EJ Haagsman HP. Interspecies Cathelicidin Comparison Reveals Divergence in Antimicrobial Activity, TLR Modulation, Chemokine Induction and Regulation of Phagocytosis. Sci Rep (2017) 7:40874. 10.1038/srep40874 28102367 PMC5244392

[20] Gonser S Crompton NE Folkers G Weber E. Increased Radiation Toxicity by Enhanced Apoptotic Clearance of HL-60 Cells in the Presence of the Pentapeptide Thymopentin, Which Selectively Binds to Apoptotic Cells. Mutat Res (2004) 558(1-2):19–26. 10.1016/j.mrgentox.2003.10.010 15036115

[21] Hu X Zhao M Wang Y Wang Y Zhao S Wu J. Tetrahydro-Beta-Carboline-3-Carboxyl-Thymopentin: A Nano-Conjugate for Releasing Pharmacophores to Treat Tumor and Complications. J Mater Chem B (2016) 4(8):1384–97. 10.1039/c5tb01930c 32263105

[22] Zhang T Qin XY Cao X Li WH Gong T Zhang ZR. Thymopentin-Loaded Phospholipid-Based Phase Separation Gel With Long-Lasting Immunomodulatory Effects: in Vitro and in Vivo Studies. Acta Pharmacol Sin (2019) 40(4):514–21. 10.1038/s41401-018-0085-8 PMC6461748 30002492

[23] Wei X Zhang L Zhang R Koci M Si D Ahmad B. A Novel Cecropin-LL37 Hybrid Peptide Protects Mice Against EHEC Infection-Mediated Changes in Gut Microbiota, Intestinal Inflammation, and Impairment of Mucosal Barrier Functions. Front Immunol (2020) 11:1361. 10.3389/fimmu.2020.01361 32695115 PMC7338479

[24] Wei XB Wu RJ Si DY Liao XD Zhang LL Zhang RJ. Novel Hybrid Peptide Cecropin a (1-8)-LL37 (17-30) With Potential Antibacterial Activity. Int J Mol Sci (2016) 17(7):983. 10.3390/ijms17070983 PMC4964367 27367675

[25] Molhoek EM van Dijk A Veldhuizen EJ Haagsman HP Bikker FJ. Improved Proteolytic Stability of Chicken Cathelicidin-2 Derived Peptides by D-Amino Acid Substitutions and Cyclization. Peptides (2011) 32(5):875–80. 10.1016/j.peptides.2011.02.017 21376095

[26] Gao D Zhang X Zhang J Cao J Wang F. Expression of Thymosin Alpha1-Thymopentin Fusion Peptide in Pichia Pastoris and Its Characterization. Arch Pharm Res (2008) 31(11):1471–6. 10.1007/s12272-001-2132-z 19023544

[27] Wang JM Wolf RM Caldwell JW Kollman PA Case DA. Development and Testing of a General Amber Force Field (Vol 25, pg 1157, 2004). J Comput Chem (2005) 26(1):114–. 10.1002/jcc.20145 15116359

[28] Maier JA Martinez C Kasavajhala K Wickstrom L Hauser KE Simmerling C. Ff14sb: Improving the Accuracy of Protein Side Chain and Backbone Parameters From Ff99sb. J Chem Theory Comput (2015) 11(8):3696–713. 10.1021/acs.jctc.5b00255 PMC4821407 26574453

[29] Pathak AK Bandyopadhyay T. Temperature Induced Dynamical Transition of Biomolecules in Polarizable and Nonpolarizable TIP3P Water. J Chem Theory Comput (2019) 15(4):2706–18. 10.1021/acs.jctc.9b00005 30849227

[30] Massova I Kollman PA. Combined Molecular Mechanical and Continuum Solvent Approach (MM-PBSA/GBSA) to Predict Ligand Binding. Perspect Drug Discov (2000) 18:113–35. 10.1023/A:1008763014207

[31] Darden T York D Pedersen L. Particle Mesh Ewald - an N.Log(N) Method for Ewald Sums in Large Systems. J Chem Phys (1993) 98(12):10089–92. 10.1063/1.464397

[32] Triantafilou M Miyake K Golenbock DT Triantafilou K. Mediators of Innate Immune Recognition of Bacteria Concentrate in Lipid Rafts and Facilitate Lipopolysaccharide-Induced Cell Activation. J Cell Sci (2002) 115(Pt 12):2603–11. 10.1242/jcs.115.12.2603 12045230

[33] Pfeiffer A Bottcher A Orso E Kapinsky M Nagy P Bodnar A. Lipopolysaccharide and Ceramide Docking to CD14 Provokes Ligand-Specific Receptor Clustering in Rafts. Eur J Immunol (2001) 31(11):3153–64. 10.1002/1521-4141(200111)31:11<3153::aid-immu3153>3.0.co;2-0 11745332

[34] Whitley NT Day MJ. Immunomodulatory Drugs and Their Application to the Management of Canine Immune-Mediated Disease. J Small Anim Pract (2011) 52(2):70–85. 10.1111/j.1748-5827.2011.01024.x 21265846

[35] Waldmann TA. Cytokines in Cancer Immunotherapy. Cold Spring Harb Perspect Biol (2018) 10(12). 10.1101/cshperspect.a028472 PMC6280701 29101107

[36] Berraondo P Sanmamed MF Ochoa MC Etxeberria I Aznar MA Perez-Gracia JL. Cytokines in Clinical Cancer Immunotherapy. Br J Cancer (2019) 120(1):6–15. 10.1038/s41416-018-0328-y 30413827 PMC6325155

[37] Mei YX Chen HX Zhang J Zhang XD Liang YX. Protective Effect of Chitooligosaccharides Against Cyclophosphamide-Induced Immunosuppression in Mice. Int J Biol Macromol (2013) 62:330–5. 10.1016/j.ijbiomac.2013.09.038 24080320

[38] Fan YP Lu Y Wang DY Liu JG Song XP Zhang WM. Effect of Epimedium Polysaccharide-Propolis Flavone Immunopotentiator on Immunosuppression Induced by Cyclophosphamide in Chickens. Cell Immunol (2013) 281(1):37–43. 10.1016/j.cellimm.2013.01.008 23435348

[39] Ren Z He CH Fan YH Guo LW Si HM Wang YW. Immuno-Enhancement Effects of Ethanol Extract From Cyrtomium Macrophyllum (Makino) Tagawa on Cyclophosphamide-Induced Immunosuppression in BALB/C Mice. J Ethnopharmacol (2014) 155(1):769–75. 10.1016/j.jep.2014.06.021 24960181

[40] Gong Y Wu J Li ST. Immuno-Enhancement Effects of Lycium Ruthenicum Murr. Polysaccharide on Cyclophosphamide-Induced Immunosuppression in Mice. Int J Clin Exp Med (2015) 8(11):20631–7. PMC4723828 26884983

[41] Yun LY Wu T Li Q Zhang M. Dietary Supplementation With Purified Wheat Germ Glycoprotein Improve Immunostimulatory Activity in Cyclophosphamide Induced Balb/C Mice. Int J Biol Macromol (2018) 118:1267–75. 10.1016/j.ijbiomac.2018.06.199 29981325

[42] Fan HJ Xie ZP Lu ZW Tan ZB Bi YM Xie LP. Anti-Inflammatory and Immune Response Regulation of Si-Ni-San in 2,4-Dinitrochlorobenzene-Induced Atopic Dermatitis-Like Skin Dysfunction. J Ethnopharmacol (2018) 222:1–10. 10.1016/j.jep.2018.04.032 29698775

[43] Andreone L Gimeno ML Perone MJ. Interactions Between the Neuroendocrine System and T Lymphocytes in Diabetes. Front Endocrinol (Lausanne) (2018) 9:229. 10.3389/fendo.2018.00229 29867762 PMC5966545

[44] Belikov AV Schraven B Simeoni L. T Cells and Reactive Oxygen Species. J BioMed Sci (2015) 22:85. 10.1186/s12929-015-0194-3 26471060 PMC4608155

[45] Fan KJ Li YW Wu J Li J Zhang J Wang QS. The Traditional Chinese Medicine Fufang Shatai Heji (STHJ) Enhances Immune Function in Cyclophosphamide-Treated Mice. Evid Based Complement Alternat Med (2020) 2020:3849847. 10.1155/2020/3849847 32063984 PMC6998758

[46] Schroeder HW Jr. Cavacini L. Structure and Function of Immunoglobulins. J Allergy Clin Immunol (2010) 125(2 Suppl 2):S41–52. 10.1016/j.jaci.2009.09.046 PMC3670108 20176268

[47] Corthesy B. Roundtrip Ticket for Secretory Iga: Role in Mucosal Homeostasis? J Immunol (2007) 178(1):27–32. 10.4049/jimmunol.178.1.27 17182536

[48] Yu Q Nie SP Wang JQ Liu XZ Yin PF Huang DF. Chemoprotective Effects of Ganoderma Atrum Polysaccharide in Cyclophosphamide-Induced Mice. Int J Biol Macromol (2014) 64:395–401. 10.1016/j.ijbiomac.2013.12.029 24370474

[49] Seong SY Matzinger P. Hydrophobicity: An Ancient Damage-Associated Molecular Pattern That Initiates Innate Immune Responses. Nat Rev Immunol (2004) 4(6):469–78. 10.1038/nri1372 15173835

[50] Park S Shin HJ Shah M Cho HY Anwar MA Achek A. TLR4/MD2 Specific Peptides Stalled in Vivo LPS-Induced Immune Exacerbation. Biomaterials (2017) 126:49–60. 10.1016/j.biomaterials.2017.02.023 28254693

[51] Zhang YL Liu ZG Wu JZ Bai B Chen HJ Xiao ZX. New MD2 Inhibitors Derived From Curcumin With Improved Anti-Inflammatory Activity. Eur J Med Chem (2018) 148:291–305. 10.1016/j.ejmech.2018.02.008 29466778

[52] Peddireddy V Doddam SN Qureshi IA Yerra P Ahmed N. A Putative Nitroreductase From the Dosr Regulon of Mycobacterium Tuberculosis Induces Pro-Inflammatory Cytokine Expression Via TLR2 Signaling Pathway. Sci Rep (2016) 6:24535. 10.1038/srep24535 27094446 PMC4837367

[53] Hayden MS Ghosh S. Signaling to NF-Kappa B. Gene Dev (2004) 18(18):2195–224. 10.1101/gad.1228704 15371334

[54] Kawai T Akira S. The Role of Pattern-Recognition Receptors in Innate Immunity: Update on Toll-Like Receptors. Nat Immunol (2010) 11(5):373–84. 10.1038/ni.1863 20404851

[55] Zhang D Cheng L Huang X Shi W Xiang J Gan H. Tetrandrine Ameliorates Dextran-Sulfate-Sodium-Induced Colitis in Mice Through Inhibition of Nuclear Factor-Kappa B Activation. Int J Colorectal Dis (2009) 24(1):5–12. 10.1007/s00384-008-0544-7 18685855

